# Petitions for Extreme Risk Protection Orders and Second Amendment Sanctuary Status in Colorado

**DOI:** 10.1001/jamanetworkopen.2024.4381

**Published:** 2024-04-01

**Authors:** Christopher E. Knoepke, Leslie M. Barnard, Nisha Batta, Megan McCarthy, Kimberly Thies, Christian Olivencia, Caitlin Robinson, Shalyn Kettering, Sheila Huss, Marian E. Betz

**Affiliations:** 1 Firearm Injury Prevention Initiative, University of Colorado School of Medicine, Aurora; 2 Division of Cardiology, University of Colorado School of Medicine, Aurora; 3 Adult and Child Center for Outcomes Research & Delivery Science, University of Colorado School of Medicine, Aurora; 4 Department of Epidemiology, University of Colorado School of Public Health, University of Colorado Anschutz Medical Campus, Aurora; 5 Injury and Violence Prevention Center, University of Colorado School of Medicine and University of Colorado School of Public Health, Aurora; 6 Colorado Office of the Attorney General, Denver, Colorado; 7 School of Public Affairs, University of Colorado, Denver; 8 Department of Emergency Medicine, School of Medicine, University of Colorado Anschutz Medical Campus, Aurora

## Abstract

**Importance:**

Extreme risk protection orders (ERPOs) temporarily bar individuals adjudicated as being at risk of violence (including suicide) from buying or possessing firearms. In protest, many US jurisdictions have declared themselves “Second Amendment sanctuaries” (2A sanctuaries). Many 2A sanctuaries continue to use ERPOs in low numbers, suggesting a poorly defined risk threshold at which they are acceptable.

**Objective:**

To characterize circumstances under which ERPOs are used in 2A sanctuaries, highlighting their most broadly acceptable applications.

**Design, Setting, and Participants:**

This cross-sectional study of civil court documents analyzed petitions for ERPOs filed in Colorado from January 2020 to December 2022. All petitions during the study period were included following de-duplication. These include petitions filed by law enforcement and family members against adults allegedly at risk of firearm violence across the state. Data were analyzed on a rolling basis between January 2020 and June 2023.

**Exposure:**

ERPO petition filed in Colorado.

**Main Outcomes and Measures:**

Seventy-seven data elements defined a priori were abstracted from all petitions and case files, including respondent demographics, petitioner types (family or law enforcement), types of threats (self, other, mass violence, combination), violence risk factors, and case outcomes (granted, denied).

**Results:**

Of a total 338 ERPOs filed in Colorado, 126 (37.3%) occurred in 2A sanctuaries. Sixty-one of these 2A petitions were granted emergency orders, and 40 were full 1-year ERPOs after a hearing. Forty ERPOs (31.7%) were petitioned for by law enforcement. Petitions in non-2A counties were more likely to have been filed by law enforcement (138 of 227 [64.9%] vs 40 of 126 [31.7%]; *P* < .001) and to have had an emergency order granted (177 of 227 [78.0%] vs 61 of 126 [48.4%]; *P* < .001) than in 2A sanctuaries. Qualitative analysis of cases in 2A sanctuaries revealed common aggravating risk characteristics, including respondents experiencing hallucinations, histories of police interaction, and substance misuse. ERPOs have been granted in 2A sanctuaries against individuals threatening all forms of violence we abstracted for (themselves, others, and mass violence).

**Conclusions and Relevance:**

In this examination of ERPO petitions across Colorado, more than a third of filings occurred in 2A sanctuaries. Nonetheless, law enforcement represent proportionately fewer petitions in these areas, and petitions are less likely to be granted. Serious mental illness, substance misuse, and prior interactions with law enforcement featured prominently in 2A sanctuary petitions. These case circumstances highlight dangerous situations in which ERPOs are an acceptable risk-prevention tool, even in areas politically predisposed to opposing them.

## Key Points

**Question:**

What are the circumstances under which extreme risk protection orders (ERPOs) have been used in counties that have made political proclamations against their use (Second Amendment [2A] sanctuaries)?

**Findings:**

In this cross-sectional analysis of all 338 ERPO petitions filed in Colorado from 2020 to 2022, 37.3% were filed in 2A sanctuaries, with 31.7% of these petitions were made by law enforcement: cases included respondents with serious mental health and/or substance use problems; histories of interaction with police; and threats of mass violence, violence against specific others against law enforcement, and suicide. When compared with ERPO petitions filed in non-2A sanctuary counties, those from sanctuaries were less likely to have been petitioned for by law enforcement and less likely to be granted.

**Meaning:**

2A sanctuaries are seen as a barrier to ERPO use, but there exist several case characteristics that seem to overcome political reticence to use ERPOs; petitioners should be more assured in these cases that petitions will be considered, irrespective of the local political climate.

## Teaser

This cross-sectional study examines the relative frequency and characteristics of extreme risk protection order petitions filed in areas of Colorado that have declared themselves Second Amendment sanctuaries.

## Introduction

Extreme risk protection orders (ERPOs) are a risk-based legal tool designed to help prevent firearm violence. ERPOs (also colloquially referred to as “red flag laws”) are civil restraining orders that allow eligible individuals or groups (“petitioners”) to formally request a judge to temporarily prohibit specific individuals (“respondents”) from purchasing or otherwise possessing firearms, so long as the judge agrees that the respondent is contemporaneously at extreme risk of firearm violence.^1,2,3^ While processes vary slightly by state, ERPOs are typically reviewed by a judge for an immediate emergency ex parte order, which is reviewed at a hearing to determine if the order would be extended to a year. As of June 2023, ERPO-type laws had been implemented in 21 states and the District of Columbia. In addition to being a promising suicide prevention tool,^4^ ERPOs have been used in hundreds of cases of threatened mass shootings, including both public mass shooting threats (directed at schools, workplaces, health care facilities, etc) and private threats directed toward specific individuals, most commonly respondents’ family members and romantic partners.^1,2,5,6^

By focusing firearms access restrictions to individuals demonstrating high risk of violence, ERPOs seek to reduce the collective impact of violence without burdening the vast majority of firearm owners who do not pose such a risk. While seemingly politically divisive, ERPOs enjoy high levels of support across the general population.^7,8^ A 2019 survey by American Public Media showed widespread support for law enforcement and family-initiated ERPOs across subgroups, including gender, region, educational status, political affiliation and whether the person owned a gun or otherwise lived in a home with firearms.^9^ However, political opposition to ERPOs has manifested in several states through the creation of self-declared Second Amendment sanctuary (2A sanctuary) areas. Firearms rights advocates estimate that more than 1400 US counties have made 2A sanctuary declarations.^10^ Sanctuary declarations are typically made by county commissions or other elected bodies, but they do not actually prohibit petitioning for or granting ERPOs in these jurisdictions. However, ERPOs have been petitioned for at a lower rate in Colorado’s 2A counties than in those that did not make such a declaration.^5^ Lower rates of petitioning in 2A sanctuary jurisdictions does not mean that risk of firearm violence is lower at the population level; in fact, recent analyses suggest that rates of firearm-related deaths are higher in rural areas (where 2A sanctuary counties are concentrated) than in urban ones.^11^ Judges in Colorado and elsewhere are allowed wide discretion in granting or denying petitions they review. Statutory guidance provided to them includes the directive to include “any relevant evidence” alongside a nonexhaustive list of potentially aggravating case circumstances (including respondent’s documented history of violence or threats, access to firearms, substance misuse, etc). In many cases, then, whether an ERPO is filed may be partially a function of whether the community in which the at-risk person resides is a 2A sanctuary or not. However, the fact that there have been petitions and granted orders in 2A sanctuaries suggests that a poorly defined threshold of risk exists that overrides political resistance. In these cases, law enforcement or other petitioners exercise the option to petition for an ERPO due to concern for community or individual safety.

### Colorado’s ERPO Law and Second Amendment Sanctuaries

Colorado provides a useful laboratory in which to better understand the dynamics underlying the utility of ERPOs as a population-wide violence and injury prevention tool. The state has a moderate political climate, where more densely populated urban centers exert legislative power over areas of the state that, while less populated, includes a heterogenous mix of rural, mountain, resort, agricultural, and frontier communities outside the Interstate 25 corridor. Colorado also has the seventh highest suicide rate in the US and an extensive history of high-profile public mass shootings that often serve as the political impetus to pass firearm safety legislation such as ERPOs.^12^ This dynamic was exemplified by the passage of the Deputy Zachari Parrish III Violence Prevention Act, which created Colorado’s ERPO system after multiple unsuccessful attempts at passing similar legislation. Deputy Parrish was killed and several colleagues seriously injured during a call for service on New Years Eve 2017 while attempting to de-escalate an individual with an extensive history of concerning behavior. This included several prior contacts with law enforcement due to episodes of psychosis, concern expressed by family members, and threats posted to social media (including those directed at law enforcement), alongside possession of multiple firearms, including at least 2 high-velocity semiautomatic rifles. Deputy Parrish’s eponymous legislation was signed into law in 2019 and the first petitions were filed in January 2020.

During the period following passage of the legislation but prior to the first ERPO petitions being filed, 37 of Colorado’s 64 counties declared themselves 2A sanctuaries. These declarations were similar to those seen the year prior in Illinois, where dozens of rural, politically conservative counties adopted language used contemporaneously by politically liberal jurisdictions in refusals to prioritize immigration enforcement (ie, sanctuary cities).^13^ These 37 counties are largely rural, with the addition of El Paso, Weld, and Douglas Counties (where Deputy Parrish was killed) located along Colorado’s more populous Interstate 25 corridor. As has been observed in other states with these laws, early uptake of ERPOs was heterogenous across Colorado, with pronounced differences observed in 2A sanctuary counties. Thirty-three counties had zero petitions in 2020, including 28 2A sanctuaries, and the rate of petitions per 100 000 residents was only 1.52, in 2A counties compared with 2.05 in counties that had not made any declaration.^5^

Circumstances that cross the undefined threshold of resistance to filing petitions have not been characterized. The goal of this study was to clarify these circumstances, shedding light on the situations in which ERPOs are even more acceptable as a form of risk-based violence prevention. In such situations, those who either work to effectuate an ERPO petition or who petition for ERPOs themselves can be more assured that efforts to limit firearms access to those at risk will be acted upon.

## Methods

All data gathering and analyses were approved by the Colorado Multiple institutional review board. Reporting was guided by and adhered to the Strengthening the Reporting of Observational Studies in Epidemiology (STROBE) reporting guideline, and all data are available upon request to study investigators.

We examined data from every case record related to an ERPO petition in Colorado between January 1, 2020, and December 31, 2022, which were obtained using a combination of public records request strategies. A set of 77 predefined data elements (including county of jurisdiction, case circumstances, petitioner type, and disposition, plus an overarching free response case summary) were abstracted from each individual petition by a team of trained researchers using an approach that has been previously described.^2,5^ The entire abstraction team (L.B., M.M., N.B., C.R., K.T., C.O., C.K.) met on a biweekly basis to discuss questions related to abstraction process and data quality, and a random 10% subsample of all cases were reviewed by a senior researcher (L.B.) to ensure consistent abstraction. Petitions filed in 2A sanctuary counties (defined according to self-declared 2A sanctuary status as of December 31, 2019) were included for analysis. Case summaries for petitions filed in these counties were analyzed to identify representative cases. We tested separately for differences between 2A and non-2A petitions in terms of respondent demographics and ERPO outcomes. We used χ^2^ for binary categorical variables and ANOVA for categorical variables with multiple levels; an α = .05 was used for significance testing. All statistical analyses were conducted using SAS version 9.4 (SAS Institute Inc).

## Results

During the study period, 353 ERPO petitions were filed across Colorado—96 in 2020, 104 in 2021, and 153 in 2022 (Table). Among these petitions, 126 (36.8%; 30 in 2020, 36 in 2021, and 59 in 2022) were filed in 20 of the state’s 37 2A sanctuary counties; the remaining 17 2A sanctuary counties did not have any ERPOs filed during the study period. Sixty-one of these petitions from 2A sanctuary counties resulted in emergency orders (TERPOs), 40 of which led to full 1-year ERPOs after a hearing. ERPO petitions in non-2A sanctuaries were both more likely to be petitioned for by law enforcement (138 of 227 [64.9%] vs 40 of 126 [31.7%]; *P* < .001) and to have been granted a TERPO (177 of 227 [78.0%] to 61 of 126 [48.4%]; *P* < .001) than those in 2A sanctuary counties. ERPOs have been filed in 2A sanctuaries against individuals for threats of suicide only (8 [7.8%]), targeted violence against family members or law enforcement (95 [92.2%]), and for threatening mass violence (ie, threatening to shoot 3 or more people, not including 18 individual who were threatening themselves [17.5%]).

**Table.** Respondent and Case Characteristics for ERPOs filed in Colorado 2A Sanctuary and Non-2A Sanctuary Counties, 2020-2022

Variable	ERPOs, No. (%)		*P* value
	2A counties (n = 126)	Non-2A counties (n = 227)	
**Respondent demographic characteristics**			
Age^a^			
18-30 y	26 (26.3)	55 (27.8)	.84
31-39 y	27 (27.3)	54 (27.3)	
40-59 y	27 (27.3)	59 (29.8)	
≥60 y	19 (19.2)	30 (15.2)	
Gender^b^			
Female	24 (19.4)	28 (12.3)	.09
Male	100 (80.6)	197 (86.8)	
Race^c^			
American Indian or Alaska Native	0	0	NA
Asian	1 (1.4)	4 (1.8)	.42
Black	3 (4.3)	15 (6.6)	.047
Native Hawaiian or Pacific Islander	0	0	NA
White	65 (94.2)	161 (70.9)	.003
Other	2 (2.9)	7 (3.1)	.47
Hispanic ethnicity^d^			
Yes	6 (8.7)	4 (1.8)	.02
No	63 (91.3)	196 (86.3)	
**Case characteristics and outcomes**			
TERPO Status			
Granted	61 (48.4)	177 (78.0)	<.001
Denied or dismissed	65 (51.2)	50 (22.0)	
ERPO status			
Granted	40 (65.6)	127 (71.8)	.36
Denied or dismissed	21 (34.4)	50 (28.2)	
Petitioner			
Law enforcement	40 (31.7)	138 (64.9)	<.001
Non–law enforcement	86 (68.3)	75 (35.2)	
Filed for threats^e^			
Self	8 (7.8)	34 (17.4)	.06
Others	72 (69.9)	116 (59.5)	
Both	23 (22.3)	45 (23.1)	

Abbreviations: ERPO, extreme risk protection order; TERPO, emergency ERPO.

^a^ Data missing for 27 in 2A counties and 15 in non-2A counties.

^b^ Data missing for 4 in 2A counties.

^c^ More than 1 category possible. Data missing for 57 in 2A counties and 57 in non-2A counties.

^d^ Data missing for 57 in 2A counties and 27 in non-2A counties.

^e^ Data missing for 23 in 2A counties and 32 in non-2A counties.

Aggravating risk characteristics commonly occurring in cases where ERPOs were granted in 2A sanctuaries included respondents’ experiencing hallucinations, having a history of interaction with police, and current acute substance misuse (eAppendix in Supplement 1). In several cases, petitioners abandoned their pursuit of ERPOs in favor of other civil restraining orders, including those that also prohibit possession of firearms. These cases were included in this analysis and are included in the accompanying series of representative cases.

## Discussion

Despite political declarations against their use, a lower proportion of law enforcement petitions and granting of emergency orders when compared with non-2A sanctuary jurisdictions, utilization of ERPO petitions in 2A sanctuary counties is not zero. Cases in which petitions were filed serve to highlight factors with more universal acceptability of risk-based firearms access interventions. This acceptability of ERPOs extended to the judicial systems in these counties, as approximately half of all petitions were granted an emergency order, and a third resulted in a year-long ERPO. In particular, cases where the respondent suffered from positive psychiatric symptoms (particularly hallucinations, irrespective of etiology) and/or had documented frequent contact with law enforcement appeared to provide an acceptable factual grounding for an ERPO petition in 2A sanctuary counties. Cases with evidence or threats of domestic violence were also present among granted ERPO petitions in these jurisdictions, as were ones in which the respondent had not threatened others but had made explicit threats of suicide (including threatening to provoke law enforcement into killing the respondent).

Additionally, several cases were identified in which temporary orders were granted, but year-long ERPOs were not pursued in favor of other civil protection orders, which can be more restrictive than ERPOs by prohibiting all forms of contact, last at least 2 years, and can become permanent in certain circumstances. It may be possible that similar courses of action are pursued in non-2A sanctuary jurisdictions, but it may also be possible that more general restraining orders are preferred in areas where ERPOs are more politically controversial.

The proportion of petitions filed by law enforcement we observed in 2A sanctuary counties (31.7% of total) was notably low. Previous studies in ERPO states with multiple petitioner groups have observed proportions of petitions from law enforcement ranging from 65% in Oregon^14^ to more than 95% in California,^15^ including 60.5% of all petitions across Colorado in 2020.^5^ Further investigation is necessary to determine the cause of these lower rates of law enforcement–initiated petitions. Possibilities include that 2A sanctuary declarations give law enforcement officers a false impression that filing for ERPOs is not legally possible in their jurisdictions, agency policies that discourage filing in light of declarations, and compensatory petitioning among other eligible petitioners (eg, family members who file under the impression that law enforcement are not able or willing to do so).

### Limitations

This report has several limitations. Colorado has only had ERPOs available since January 1, 2020, and the number of petitions and jurisdictions in which they have been filed is increasing over time. Colorado also recently expanded the list of approved petitioners to include medical and mental health professionals as well as educators,^16^ which deserves further study. Petitioning behavior among health professionals may differ from those of law enforcement and family or household members, including in 2A sanctuary counties. Colorado’s unique history of public mass violence may also bias views toward risk-based violence prevention policies in ways that are not representative of other states. Finally, the inductive nature of the narrative review leaves open the possibility that other risk characteristics are common in 2A sanctuary petitions, and that it was impossible to quantitatively characterize the presence or absence of each risk characteristic across cases.

## Conclusions

This cross-sectional analysis of ERPO use in Colorado highlights the utility of ERPOs, including in geographic areas that have made political proclamations against their use. People exhibiting an extreme risk of violence (directed toward themselves, family members, partners, or groups of people) are not bound by geography. No matter the political leaning of their community, our results suggest that people facing direct or ongoing threats of gun violence often elect to pursue civil protection orders in many of the types of cases for which they were designed. As other community members outside law enforcement become more involved in ERPO petitioning (including direct petitioning), they should know that local political proclamations do not necessarily limit the legal tools available to reduce risk of violence.

## Notes

Supplement 1. **eTable.** Representative Case Summaries for ERPOs Granted in 2A Sanctuaries, Including Aggravating Risk Characteristics and Geographic Region Within Colorado

Supplement 2.
Data Sharing Statement
