# Supplementary material for: Petitions for Extreme Risk Protection Orders and Second Amendment Sanctuary Status in Colorado
Source: JAMA Netw Open. 2024 Apr 1;7(4):e244381. doi: 10.1001/jamanetworkopen.2024.4381 (PMC10985551; doi:10.1001/jamanetworkopen.2024.4381)
Supplement: Supplement 1. — eTable. Representative Case Summaries for ERPOs Granted in 2A Sanctuaries, Including Aggravating Risk Characteristics and Geographic Region Within Colorado [file jamanetwopen-e244381-s001.pdf]

Supplemental Online Content

Knoepke CE, Barnard LM, Batta N, et al. Petitions for extreme risk protection orders and Second Amendment sanctuary status in Colorado. *JAMA Netw Open.* 2024;7(4):e244381. doi:10.1001/jamanetworkopen.2024.4381

eTable. Representative Case Summaries for ERPOs Granted in 2A Sanctuaries, Including Aggravating Risk Characteristics and Geographic Region Within Colorado

This supplemental material has been provided by the authors to give readers additional information about their work.

© 2024 Knoepke CE et al. *JAMA Network Open.*

eTable. Representative Case Summaries for ERPOs Granted in 2A Sanctuaries, Including Aggravating Risk Characteristics and Geographic Region Within Colorado				
**Threat Type(s)**	**Aggravating Case Characteristics**	**Colorado Region**	**Case narrative/summary**	**Disposition**
Threats to others	Hallucinations/neuro degenerative illness<br><br>Current substance abuse<br><br>DV threat	**Front Range**	A respondent in their 60's was taken into custody on suspicion of DUI and impersonating a police officer. The respondent made numerous delusional claims, including that they had been shot multiple times without receiving medical care, and served in the Special Forces (when police could not locate any record of military service). When they were pulled over, police found the respondent in possession of a Sheriff's uniform. Police were able to determine that the respondent had a history of being treated for unspecified mental health conditions, was currently using alcohol and and methamphetamine heavily, and documented threats directed toward their former spouse and coworkers. A total of 59 firearms and 50,000 rounds of ammunition were recovered by law enforcement.	Granted
Threats to others<br><br>Case 139	Hallucinations/neuro degenerative illness<br><br>History of interaction with police	**Southwest**	A respondent in their 70s who had been diagnosed with dementia made several claims to family caregivers that there were "people under the house trying to steal the water". These family members and other community members had called the police regarding the respondent's concerning behavior. Family members additionally reported to police that, while there were currently no firearms in their possession, the respondent was adamant about buying one in order to "kill these people".	Granted
Threats to others	Hallucinations/neuro degenerative illness<br><br>History of interaction with police<br><br>Current substance abuse	**Northwest**	An unhoused respondent with a reported extensive history of substance misuse and mental illness with psychotic features was involved in an altercation in which they threatened to kill police officers, after which they were arrested . Multiple firearms and over 1000 rounds of ammunition were found in the car they had been using for shelter. Subsequent to this arrest, the respondent was placed on an M1 involuntary psychiatric hold at a nearby hospital, and	Granted

© 2024 Knoepke CE et al. *JAMA Network Open.*

			law enforcement elected to additionally petition for an ERPO to further limit access to firearms following discharge from the hospital.	
Threats to others	DV threats<br><br>Current substance abuse	**Southern**	A respondent in their 40s with multiple previous charges of domestic violence stemming from abuse toward their daughter and former spouse. After a recent episode in which they hit and threatened the former spouse at their home, the victim filed for an ERPO. The petition noted that the respondent had been using especially heavy amounts of unspecified drugs and alcohol, and that they owned a semiautomatic rifle.	Granted
Threats to othres	DV threats<br><br>Current substance abuse	**Front Range**	An individual in their 30s had a petition filed against them by their spouse, who accused them of being physically violent and brandishing firearms to threaten and intimidate family members. The spouse noted that the respondent was particularly prone to violence while under the influence of unspecified drugs and alcohol.	Granted
Threats to self and others	Suicide threat<br><br>Current substance abuse	**Northwest**	A law enforcement officer currently experiencing heavy alcohol misuse made threats of suicide to their spouse and children. In these episodes, the respondent reportedly put a handgun in their mouth and tell their children that they planned to die. THe respondent's spouse contacted law enforcement, who subsequently filed for an ERPO ahead of their release from jail approximately a week later.	Granted
Threats to self and others	Suicide threat<br><br>Current substance abuse<br><br>Vacated for more expansive prohibition	**Southwest**	After being pulled over for a suspected DUI, the respondent ran away and into their home, screaming that they would get their gun. The individual proceeded to tell law enforcement that they would shoot themselves ad responding officers, threatened to kill herself and the officer (which was interpreted as a threat of suicide by police). The respondent had a history of alcohol misuse and was intoxicated during this altercation. One firearm was found in the home and was taken into evidence when the individual was arrested. The police filed for an ERPO, which was	Granted

© 2024 Knoepke CE et al. *JAMA Network Open.*

			granted. The ERPO was later vacated when a criminal protection order prohibiting this respondent from possessing firearms or ammunition was in place.	
Threats to others	Mass shooting threat	**Southern**	Law enforcement received an anonymous tip claiming that a teenager had been talking in positive terms about school shootings and carrying firearms at work (which was not required). They recently dropped out of the local high school following years of educational trouble. Two firearms were found and collected by law enforcement, who then filed a petition which was issued.	Granted
Threats to self and others	Mass shooting threat<br><br>Current substance abuse	**Front Range**	A person int heir 30s had been talking about killing police officers, made anti-government comments on social media, and threatened to shoot up the electric company after the utilities had been shut off, and their family reported these concerns to police. The family noted that the respondent had a long history of unspecified mental illness and substance misuse, Law enforcement contacted them and made an arrest . A total of 9 firearms were collected during the arrest, and law enforcement petitioned for an ERPO, which was granted.	Granted
Threats to others	DV threat<br><br>History of interaction with law enforcement	**Front Range**	The respondent was an individual in their 20s with an extensive history of law enforcement interaction, incarceration, and violating civil restraining orders. During their most recent incarceration, their ex-spouse filed a petition to prevent access to firearms after they were released.	Granted
Threats to others<br><br>Case 108	DV threat<br><br>Vacated for more expansive prohibition	**Front Range**	A person in their 30s had a petition filed against them by their former partner after allegedly pointing a handgun at the partner's family. After the TERPO was granted, but before the ERPO hearing, the ex-partner chose to file a civil protection order instead.	TERPO granted Denied full ERPO for CPO
Threats to others	DV threat<br><br>Current substance abuse<br><br>Vacated for more	**Western**	The respondent, an individual in their 30s, was alleged by their spouse to have threatened and perpetuated violence against them. The petition stated that the respondent owned a firearm, was	Dismissed TERPO, filed CPO

© 2024 Knoepke CE et al. *JAMA Network Open.*

	expansive prohibition		diagnosed with bipolar disorder and abused alcohol. After the TERPO was granted, the spouse was counselled to pursue a more expansive civil protection order. After filing for the CPO, the ERPO petition was dismissed by the court.	

© 2024 Knoepke CE et al. *JAMA Network Open.*
