# Supplementary material for: Petitions for Extreme Risk Protection Orders and Second Amendment Sanctuary Status in Colorado
Source: JAMA Netw Open. 2024 Apr 1;7(4):e244381. doi: 10.1001/jamanetworkopen.2024.4381 (PMC10985551; doi:10.1001/jamanetworkopen.2024.4381)
Supplement: Supplement 2. — Data Sharing Statement [file jamanetwopen-e244381-s002.pdf]

Data Sharing Statement

Knoepke. Petitions for Extreme Risk Protection Orders and Second Amendment Sanctuary Status in Colorado. *JAMA Netw Open*. Published April 01, 2024.
doi:10.1001/jamanetworkopen.2024.4381

Data
Data available: Yes
Data types: Deidentified participant data
How to access data: christopher.knoepke@cuanschutz.edu
When available: With publication

Supporting Documents
Document types: None

Additional Information
Who can access the data: Researchers whose proposed uses have been approved under a data use agreement
Types of analyses: For any research purpose
Mechanisms of data availability: With a signed data use agreement
Any additional restrictions: None
